# Surgical treatment of patellar dislocation: A network meta-analysis of randomized control trials and cohort studies

**DOI:** 10.3389/fsurg.2023.1003796

**Published:** 2023-03-30

**Authors:** Mingqing Fang, Zijun Cai, Linyuan Pan, Yilan Ding, Yueyao Zhang, Siyuan Cheng, Yifan Wang, Jialin Gao, Yusheng Li, Wenfeng Xiao

**Affiliations:** ^1^ Department of Orthopedics, Xiangya Hospital, Central South University, Changsha, China; ^2^ National Clinical Research Center for Geriatric Disorders, Xiangya Hospital, Central South University, Changsha, China; ^3^ Xiangya School of Medicine, Central South University, Changsha, China

**Keywords:** network meta-analysis, medial patellofemoral ligament reconstruction, medial soft tissue surgery, single bundle, double bundle, patellar dislocation

## Abstract

**Background:**

Currently, there are many surgical options for patellar dislocation. The purpose of this study is to perform a network meta-analysis of the randomized controlled trials (RCTs) and cohort studies to determine the better treatment.

**Method:**

We searched the Pubmed, Embase, Cochrane Central Register of Controlled Trials, Web of Science, clinicaltrials.gov and who.int/trialsearch. Clinical outcomes included Kujala score, Lysholm score, International Knee Documentation Committee (IKDC) score, redislocation or recurrent instability. We conducted pairwise meta-analysis and network meta-analysis respectively using the frequentist model to compare the clinical outcomes.

**Results:**

There were 10 RCTs and 2 cohort studies with a total of 774 patients included in our study. In network meta-analysis, double-bundle medial patellofemoral ligament reconstruction (DB-MPFLR) achieved good results on functional scores. According to the surface under the cumulative ranking (SUCRA), DB-MPFLR had the highest probabilities of their protective effects on outcomes of Kujala score (SUCRA 96.5 %), IKDC score (SUCRA 100.0%) and redislocation (SUCRA 67.8%). However, DB-MPFLR (SUCRA 84.6%) comes second to SB-MPFLR (SUCRA 90.4%) in Lyshlom score. It is (SUCRA 70%) also inferior to vastus medialis plasty (VM-plasty) (SUCRA 81.9%) in preventing Recurrent instability. The results of subgroup analysis were similar.

**Conclusion:**

Our study demonstrated that MPFLR showed better functional scores than other surgical options.

## Introduction

Patellar dislocation is a serious injury, accounting for 3.3% of all knee injuries, and females aged 10–17 are at the highest risk (1, 2). Failure or suboptimal treatment may result in serious problems, such as recurrent instability, keen pain, and even osteoarthritis. Those with a history of dislocation were seven times more likely to have an unstable episode during follow-up than those with a first dislocation (3). Therefore, appropriate and effective treatments are urgently needed.

A meta-analysis has reported that conservative treatments can be used for patients with lower risk, while surgery should be considered for patients with higher risk (1). However, there are different surgical options, such as medial retinaculum plication (MR-plication), medial retinaculum plasty (MR-plasty), VM-plasty, medial capsule reefing (MC-reefing), medial patellofemoral ligament reconstruction (MPFLR) and so on. In addition, a study has shown that approximately from the femoral origination point, the medial patellofemoral ligament (MPFL) consists of two relatively concentrated fiber bundles: the inferior-straight bundle and the superior-oblique bundle (4). Thus, two different methods of reconstructing the medial patellar ligament were proposed, single-bundle MPFLR (SB-MPFLR) and DB-MPFLR respectively. However, there is no standard consensus on surgical options for the treatment of patellar dislocation.

This study aimed to perform a network meta-analysis of RCTs and cohort studies in the literature to clarify differences in surgical options and provide evidence for the better treatment. The hypothesis is that DB-MPFLR would repair the medial soft tissue structure better compared with other options.

## Materials and methods

### Study selection

This study was according to Cochrane Review methods, and reported based on the Preferred Reporting Items for Systematic Reviews and Meta-Analyses (PRISMA) guidelines. Two reviewers independently performed a literature search, reviewing the titles and abstracts of all results, and then conducting a full-text review. We manually screened all references in the study and all literature reviews found in the search results to find articles that met the inclusion criteria. We used some combined text and MeSH terms (“patellar dislocation”, “medial patellofemoral ligament reconstruction”, “plication”, “plasty”, and “reefing”) to search the Cochrane Central Register of Controlled Trials, Pubmed, Embase, Web of Science, clinicaltrials.gov and who.int/trialsearch. The complete search used for electronic databases was listed in Supplementary Appendix A. This search was carried out in December 2021.

### Inclusion criteria

(1) Human studies; (2) Studies that evaluated clinical outcomes of MPFLR or other soft tissue surgeries (plication, plasty, reefing etc.); (3) RCTs or cohort studies; (4) Published in English language; (5) Studies reporting at least one of the following data: Kujala score, Lysholm score, IKDC score, redislocation or recurrent instability.

### Exclusion criteria

(1) Subjects with knee disease or previous knee surgery; (2) Studies that only reported preoperative or intraoperative outcomes.

### Data collection and analysis

The studies were independently evaluated by two authors, followed by full-text readings of potentially eligible articles for eventual inclusion. The uncertainties included in the study were resolved through discussion and negotiation. Eligible data were extracted independently by one author into a pre-defined format and then extracted by another author for a second time to ensure accuracy. We collected information concerning (1) study characteristics including journal, authors, year of publication, study design, and level of evidence; (2) demographics of patients including the number of subjects, gender, age, surgical techniques, postoperative rehabilitation, and duration of follow-up. (3) the outcomes of studies including the Kujala score, Lysholm score, IKDC score, redislocation, or recurrent instability.

### Assessment of risk of bias and quality of evidence

The assessment is done independently by two investigators using the Cochrane Risk of Bias Tool for RCTs, while the cohort studies were assessed using the modified Newcastle-Ottawa Scale (NOS) (5, 6). Any disagreement between the two authors was resolved through discussion and, if no agreement could be reached after discussion, it was left to the judgment of the third author.

### Statistical analysis

We conducted a pairwise meta-analysis and a network meta-analysis in a frequentist model (7). In addition, we performed a subgroup analysis of recurrent patellar dislocation in network meta-analysis. The relative effect sizes of continuous outcomes in data analysis were mean difference (MD) with confidence interval (CI) of 95% and odds ratios (ORs) and 95% CIs were calculated to evaluate the dichotomous outcomes. The level of statistical significance was set as *p* < 0.05. All statistical analyses were performed using R 4.1.1. The I-Squared (*I*^2^) test was used to estimate heterogeneity among studies for pairwise meta-analyse and network meta-analysis (8). According to the Cochrane Collaboration Handbook, only if *I*^2 ^< 25%, the heterogeneity between studies is low. Therefore, in the heterogeneity test, if *I*^2^ < 25%, fixed-effects model was adopted; otherwise, we conducted the random-effects model. For the network meta-analysis, the analysis was performed in a frequentist model employing the “netmeta” packages. For each outcome, we used a trail network plot to show the comparison of all interventions. SUCRA was used to represent the overall ranking of an intervention; that was, the higher the value of SUCRA, the higher the probability of this surgical option being the best intervention (9). We calculated the value of SUCRA to rank each intervention. Publication bias of the studies was assessed by observing the symmetry of comparison-adjusted funnel plots.

### CINeMA assessment

The confidence in network meta-analysis (CINeMA) framework was used to assessed the certainty of the evidence (10). The CINeMA evaluation consists of six evaluable items: within-study bias, reporting bias, indirectness, imprecision, heterogeneity, and incoherence. There are four levels of evidence: high, medium, low, and very low. The grade of RCT is high and The grade of cohort studies is low before evaluation.

## Results

### Results of the search

**Figure 1:**
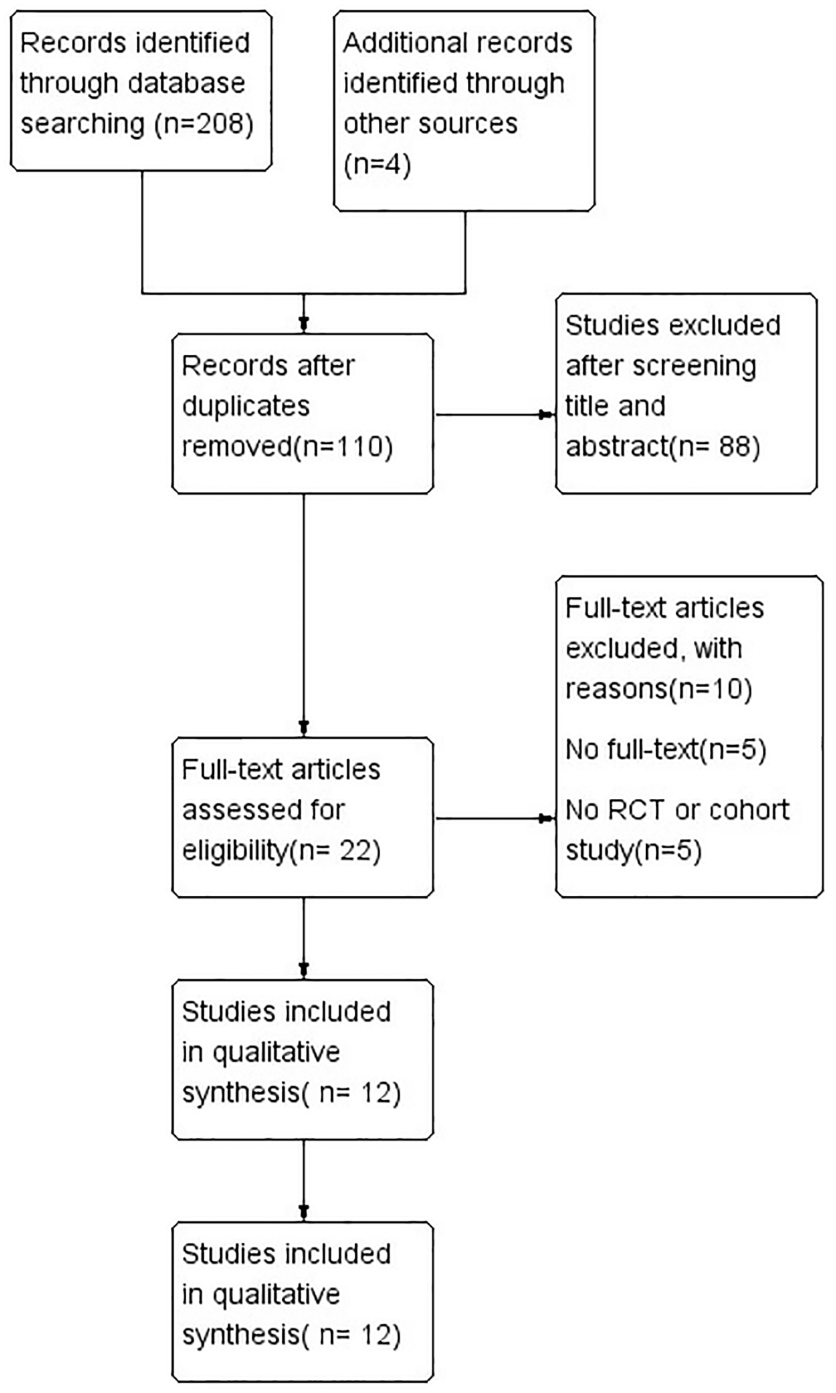
Search flow diagram. The search flow diagram summarizes the search, screening, retrieval, and appraisal of articles finally included in the network meta-analysis.

A total of 208 articles were queried from the databases with additional 4 records identified through other sources. Of these, 102 were duplicates in the databases and were subsequently excluded. The remaining 110 papers were carefully screened for titles and abstracts, and only 22 remained, excluding all others as irrelevant to the purpose of the study. We reviewed the full text of the remaining 22 articles and subsequently excluded 10 due to the lack of necessary data. Ultimately, a total of 10 RCTs and 2 cohort studies were included for data extraction and meta-analysis. Figure 1 illustrates the process of systematic literature retrieval and research selection.

### Characteristics of the included studies

This review includes 12 trials involving 774 patients. These studies were published between 2011 and 2020. A total of 6 different surgical methods were introduced for the 10 RCTs and 2 cohort studies. Of the included 12 studies, 12 studies provided Kujala scores, 6 provided Lysholm scores, 5 provided IKDC scores and 9 provided redislocation and 8 provided recurrent instability respectively. The sample sizes of the included trials ranged from 45 to 88 patients, with the mean ages ranging from 13 to 29 years and the duration of follow-up ranging from 24 to 87 months. The basic characteristics of the included studies are summarized in Table 1.

**Table 1:** Characteristics of the included studies.

Author	G 1	G 2	Patients (knees)		Sex (M/F)		Age in years mean (SD)		Follow-up (month)	Disease types	Additional surgeries	Postoperative Rehabilitation
			Included (G1/G2)	Assessed (G1/G2)	G1	G2	G1	G2				
Du et al. 2017 (31)	MR- plication	DB- MPFLR	22 (22)/ 23 (23)	22 (22)/ 23 (23)	NR		19.3 ± 6.5		24	RPD	LRR was performed in all patients	NR
Zhao et al.(1) 2012 (32)	MR- plication	DB-MPFLR	50(50)/50(50)	43(43)/45 (45)	7/36	8/37	23.9 ± 5.8	25.0 ± 6.6	24/ 60	RPD	Tibial tubercle anteromedialization or distalization was performed as indicated, and LRR was performed in all patients	The 2 groups followed the same rehabilitation protocol. A hinged brace was used for 6 weeks and locked in extension while walking.Partial to full weightbearing was allowed immediately after the operation. Range-of-motion exercises began immediately, but the knee flexion angle was restricted to 45°, 90°, and 120°in the first, second, and third 2-week periods after the operation, respectively. Straight-leg raising, vastus medialis exercises, and proprioception training began 6 weeks after the operation; running and agility training began after the fourth month.
Ma et al.(1) 2013 (33)	DB- MPFLR	MR- plasty	36 (36) / 34 (34)	32 (32) / 31 (31)	10/22	12/19	28.4 ± 4.2	28 ± 4	40	RPD	LRR was performed in all patients	The 2 groups followed the same rehabilitation protocol. Postoperatively, a hinged brace was used for 6 weeks and locked in extension during walking. Quadriceps-setting exercises and straight leg raising were encouraged from the first day after surgery. A hinged brace with a patellofemoral stabilizing component was used for 6 weeks after surgery, and it was locked in extension during walking. Walking with partial weight bearing on 2 crutches was also permitted as tolerated and gradually progressed until, after 4 weeks, full weight bearing was allowed and knee flexion up to 90° was obtained. After 6 weeks, with the removal of the brace, patients were advised to wear a knee pad to stabilize the patella during sports or rehabilitation exercises. Patients who achieved sufficient quadriceps strength and stability of the patella and sufficient range of motion were allowed to begin normal activities of daily living and jogging 2 months after surgery and to start to do athletic exercises 3 months after surgery.
Niu et al. 2016 (34)	DB- MPFLR	MR- plasty	26 (26) / 28 (28)	22 (22) / 22 (22)	10/12	9/13	27.46 ± 4.84	25.64 ± 3.35	12/24/48	RPD	LRR was performed on request.	The rehabilitation programme was similar in the two groups. There was a need to make the affected limb immobilized postoperatively. The first day after operation, the patients started moderate exercises, such as isometric contraction of the quadriceps, the affected limb straight rising, etc. Two days later, a slight knee flexion was admitted. And the degree of the knee flexion was gradually increasing from 0 to 90 during one month. At the third day afteroperation, the patients could walk on crutches. Three weeks later, the affected limb was allowed partially weight-bearing and full weightbearing five weeks or later after operation. And the immobilizer was removed three months later. Then, the patients participated in normal sports six months after operation.
Li et al. 2018 (35)	DB- MPFLR	SB- MPFLR	46 (46) / 45 (45)	45 (45) / 43 (43)	15/30	17/26	26.9 ± 5.36	27.3 ± 5.59	40.92 ± 7.22/ 41.32 ± 7.59	RPD	NO	The 2 groups followed the same rehabilitation protocol. The involved knee was placed in a hinged knee brace with a patellofemoral stabilizing component and locked in full extension, and weight bearing was avoided for the first 3 weeks postoperatively. The brace was then adjusted to allow a motion range from 0 to 90, and the patient was allowed touchdown weight bearing with crutches for the next 3 weeks. Full weight bearing and full range of motion with the brace unlocked were allowed at 6 weeks postoperatively. Isometric quadriceps exercises were suggested after surgery and throughout the whole immobilization period to prevent disuse atrophy. After 8 weeks, with the removal of the brace, patients were advised to wear a knee pad to stabilize the patella during sports or rehabilitation exercises. Patients were allowed to begin normal activities of daily living and some light sports at 2 months postoperatively. Functional activities including walking, jogging, and running were introduced at 3 months, and 6 months were needed for patients to return to normal sports activities.
Ercan et al. 2020 (36)	DB- MPFLR	SB- MPFLR	40 (40) / 40 (40)	40 (40) / 40 (40)	20/20	18/22	19 (14–29)	15 (10–28)	40 (24–74) / 46.5 (24–74)	RPD	NO	Following MPFL reconstruction, the same physical therapy and rehabilitation protocol was applied to all patients by the same team. Active and passive assisted full range of kneemotion was started the day after MPFL reconstruction.Weight bearing was gradually increased to full at 3 weeks postoperatively. Jogging was permitted 3 months after reconstruction. Return to sports was allowed 6 months postoperatively, following an evaluation of muscle strength.
Astur et al. 2015 (37)	DB- MPFLR	SB- MPFLR	62	28 (28)/30 (30)	30/28		28.32 (18–45 )	31.06 (18–45)	NR	PD	NO	Within first two-postoperative weeks, physical therapy consisted of pain and swelling control as well as active assist range of motion without weight bearing. Quadriceps muscle strengthening started in the third postoperative week with partial weight bearing until fourth week. Full weight bearing was allowed from week four onward. Full activitiy including participation in sports was allowed 12 weeks after surgery.
Zhao et al.(2) 2011 (38)	MR plication	VM plasty	60	28 (28) / 26 (26)	5/23	4/22	14.7 ± 1.3	15.2 ± 1.6	56.8 ± 21.5 / 59.1 ± 24.7	RPD	LRR was performed on request.	The 2 groups followed the same rehabilitation protocol. A hinged brace was used for 6 weeks and locked in extension while walking. Partial to full weightbearing was allowed immediately after operation. Range of motion exercise began immediately, but knee flexion anglewas restricted to 45°, 90°, and 120°, respectively, in the second, fourth, and sixth weeks after operation. Straight-leg raising, vastus medialis exercises, and proprioception training started 6 weeks after operation; running and agility training began from the fourth mont.
Ma et al.(2) 2012 (39)	MC reefing and(LPR release)	MR plasty and(LPR release)	40 (40) / 38 (38)	40 (40) / 38 (38)	16/24	15/23	28 (21–35)	29 (21–37)	60 (33–87)	PD	15 cases had the combination of lateral retinacular release (G 1) and 12 cases had the combination of LRR (G 2)	Quadriceps setting exercises and straight leg raising exercises were encouraged from the first day following surgery. Walking with partial weight bearing on two crutches andknee flex activities were also permitted within tolerance and gradually progressed from the day following surgery. After 1 week, knee flexion up to 30° was obtained. After 2 weeks knee flexion up to 90°was obtained. After 4 weeks, full weight bearing was allowed. Patients who achieved enough quadriceps strength, stability of patella, and sufficient range of motion were allowed to begin normal daily living and jogging 2 months after surgery and athletic exercises 3 months after surgery.
Ma et al.(3) 2012 (40)	MC reefing	MR plasty	33 (33) / 35 (35)	29 (29) / 32 (32)	13/16	12/20	13 (10–16)	14 (11–17)	50 (25–75)	PD	13 patients also received LRR(G 1) and 12 patients also received LRR (G 2).	The postoperative rehabilitation program and follow-up of the two groups were basically the same. Quadriceps setting exercises and straight leg raising were encouraged from the day following surgery. Walking with partial weight bearing on two crutches and knee flex activities were also permitted as tolerated and gradually progressed from the day following surgery, until after one week, knee flexion up to 90°was obtained. After 2 weeks, full weight bearing was allowed, patients were advised to wear a knee pad to stabilize the patella during sports or rehabilitation exercises. Patients who achieved a sufficient range of motion, quadriceps strength and stability of the patella were allowed to begin normal daily living and jogging at one month and return to full sports activity at 3 months.
Wang et al. 2013 (27)	DB- MPFLR	SB- MPFLR	37 (44)/21 (26)	37 (44)/21 (26)	16/21	7/14	23 ± 10	26 ± 7	48	PD	NR	The rehabilitation program was the same for single and double bundle cases. After the MPFL reconstruction patients wore a brace for one week. The patients began moderate exercise such as isometric contraction of quadriceps femoris muscle, straight leg raising, patella traction and mild genuflection two days after the operation. Flexion of 10° was allowed from the third day after the operation and the angle was increased gradually to 90° in a month. Two weeks after operation, the patients could start CPM exercises, and active or assisted kneejoint ROM exercises; three weeks after operation, the affected limb could withstand partial weight-bearing and full weight-bearing five weeks after operation or later. The patient could jog three months after operation and participate in normal sports activities six months after operation.
Fenget al. 2020 (14)	DB- MPFLR	MR- plasty	32 (32)/25 (25)	32 (32)/25 (25)	7/25	6/17	23.8 ± 5.4	23.9 ± 5.8	41	RPD	tibial tubercle Transfer was performed in all patients.	NR

Data are presented as mean, mean standard deviation, or mean (range) unless otherwise indicated.

NR, not reported;G1, Group1;G2, Group2; F, female; M, male; IKDC score, International Knee Documentation Committee subjective scores; DB-MPFLR, double bundle medial patellofemoral ligament; SB-MPFLR, single bundle medial patellofemoral ligament; MR-plication, medial retinaculum plication; MR-plasty, Medial retinaculum plasty; VM-plasty, vastus medialis plasty; MC-reefing, Medial Capsule Reefing. LRR, lateral retinacular release; patellar dislocation, PD; recurrent patellar dislocation, RPD.

### Quality assessment of included studies

We adopted the Cochrane Risk of Bias Tool for RCTs (RoB2.0) (the score for each bias domain was graded as representing a low, high, or unclear risk of bias) and modified Newcastle-Ottawa Scale (NOS) for cohort studies (Studies with scores of 7, 5 to 7, 3 to 5, and 0 to 2 were considered of good, fair, poor-fair, and poor quality, respectively) for methodological quality evaluation. Among 10 RCTs, 6 studies utilized sufficient random sequence generation methods. Appropriate methods of allocation concealment were described in 3 studies. None of the studies applied blindness to patients and researchers because of practical and ethical issues, which resulted in a high risk of bias. In 3 studies, the outcome measures were blinded. Other bias of five included RCTs was unclear. NOS indicated that the two cohort studies were of good quality. The risk of bias assessment for RCTs is shown in Supplementary Appendix B(a-b), and the risk of bias assessment for cohort studies is shown in Supplementary Appendix B(C).

### Clinical outcomes: quantitative analysis

####  Pairwise meta-analysis

For redislocation and recurrent instability, no pairwise meta-analysis was performed because the number of events in many studies was 0. All detailed results of pair-wise meta-analysis are shown in Table 2A–C.

**Table 2:** Results from pair-wise meta-analysis.

A: Kujala score				
Intervention	*n*	*I* ^2^	*p*-value	MDs (95% CI)
DB-MPFLR vs. MR-plication	2	6%	0	12.79 (10.95, 14.63)
DB-MPFLR vs. MR-plasty	2	94.5%	0.059	6.60 (−0.26, 13.45)
DB-MPFLR vs. SB-MPFLR	4	96%	0	5.75 (0.57, 10.91)
MR-plasty vs. MC-reefing	2	36.1%	0	3.21 (2.18, 4.24)
MR-plication vs. VM-plasty	1	–	0	−6.30 (−8.86, −3.74)
B: Lysholm score				
Intervention	*n*	*I* ^2^	*p*-value	MDs (95% CI)
DB-MPFLR vs. MR-plication	2	0%	0	16.75 (14.68, 18.83)
DB-MPFLR vs. MR-plasty	1	–	0	9.60 (8.12, 11.08)
DB-MPFLR vs. SB-MPFLR	2	61.1%	0.5	−1.38 (−5.38, 2.62)
MR-plication vs. VM-plasty	1	–	0	−8.70 (−11.54, −5.86)
C: IKDC score				
Intervention	*n*	*I* ^2^	*p*-value	MDs (95% CI)
DB-MPFLR vs. MR-plication	2	0%	0	18.01 (15.97, 20.05)
DB-MPFLR vs. SB-MPFLR	2	61.7%	0.232	3.30 (−2.11, 8.70)
MR-plication vs. VM-plasty	1	–	0	−9.30 (−12.87, −5.73)

#### Network meta-analysis

##### Kujala score

**Figure 2:**
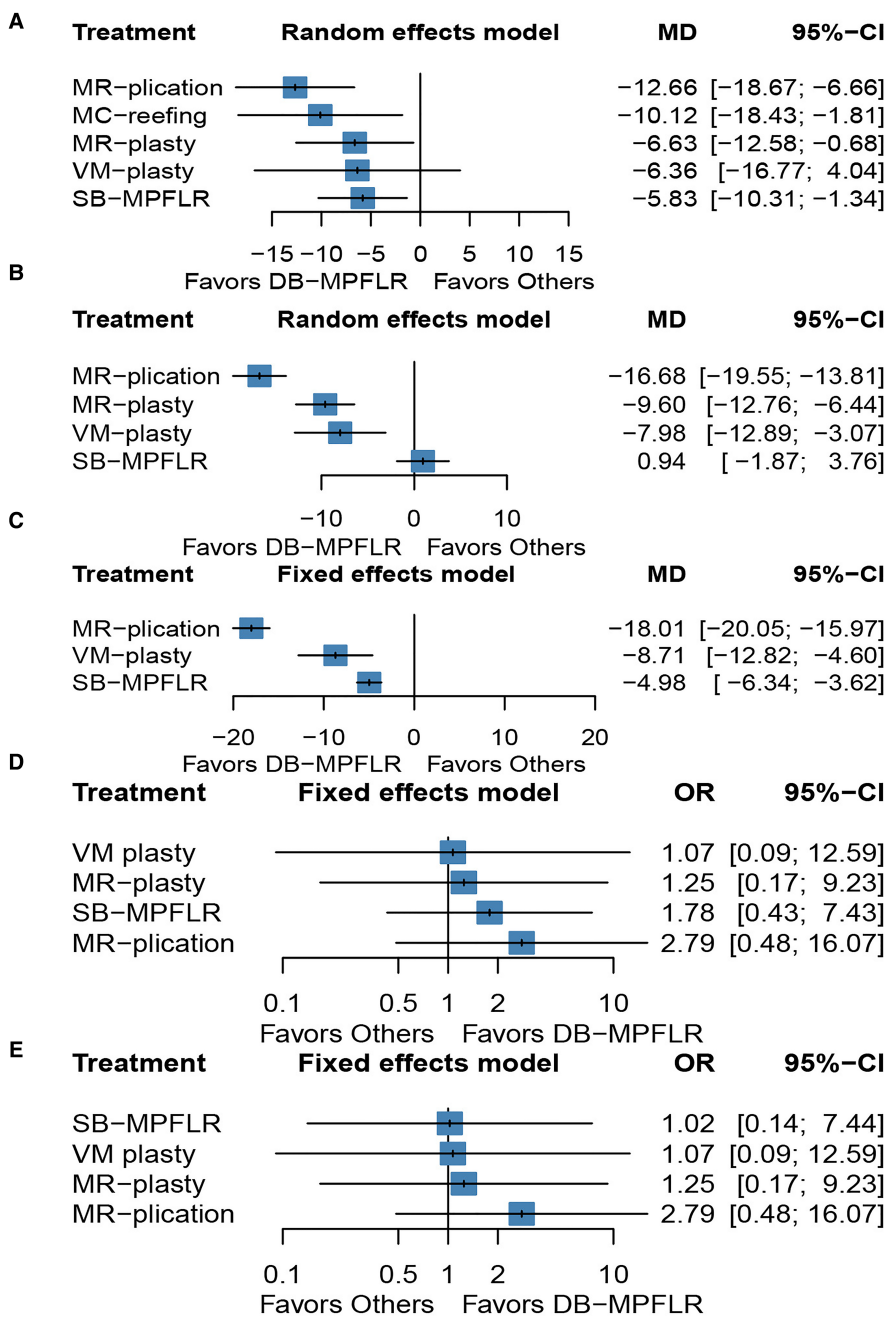
Forest plot of different surgeries: (**A**) kujala score; (**B**) lysholm score; (**C**) IKDC score; (**D**): redislocation; (**E**) recurrent instability.

Twelve included studies reported Kujala scores as one of the study outcomes. *I*^2^ = 94.1%, so we chosen the random effects model. Figure 2A shows the MD and 95% CI of each surgery compared with DB-MPFLR. MC-reefing (MD −10.12, 95% CI, −18.43, −1.81), MR-plasty (MD −6.63,95% CI, −18.43, −0.68), MR-plication (MD −12.66,95% CI, −18.67, −6.66), SB-MPFLR (MD −5.83,95% CI, −10.31, −1.34) and VM-plasty (MD −6.36,95% CI, −16.77, −4.04) were inferior to DB-MPFLR. The effects of all surgeries were ranked with SUCRA probabilities in Table 3, and DB-MPFLR had the greatest probability (SUCRA 96.5%) for being the best treatment option on Kujala score, followed by the SB-MPFLR (SUCRA 57.4%), VM-plasty(SUCRA 55.5%)and MR-plasty (SUCRA 42.0%), MC-reefing ranked in the sixth position (SUCRA 28.5%) and MR-plication ranked the last (SUCRA 9.9%).

**Table 3:** Ranking of surgeries based on probability of their protective effects on all outcomes according to the SUCRA. Larger probability, stronger protective effects.

Kujala score	Lysholm score	IKDC score	Redislocation	Recurrent instability	Subgroup of Kujala score	Subgroup of Redislocation	Subgroup of Recurrent instability	
96.5	84.6	100.0	67.8	70	95.3	65.6	67.5	DB-MPLFR
28.6	NA	NA	NA	NA	NA	NA	NA	MC-reefing
52.1	30.3	NA	55.2	56.5	38.3	55.1	56	MR-plasty
9.9	0.0	0.0	22.5	26.3	1.3	17.2	26	MR-plication
57.4	90.4	65.8	40.4	15.2	71.0	60.8	20.6	SB-MPFLR
55.5	44.6	34.2	64.2	81.9	43.9	51.3	80	VM-plasty

##### Lysholm score

Six studies reported Lysholm scores as one of the study outcomes. *I*^2^ = 42.1%, so we chosen the random effects model. Figure 2B shows the MD and 95% CI, of each surgery compared with DB-MPFLR. MR-plasty (MD −9.6,95% CI, −11.08, −8.12), MR-plication (MD −16.68,95% CI, −19.55, −13.81), and VM-plasty (MD −7.98,95% CI, −12.89, −3.07) were inferior to DB-MPFLR. DB-MPFLR was not superior to SB-MPFR (MD 0.94, 95% CI, −1.87, 3.76). As shown Table 3, SB-MPFLR had the highest probability of being the best treatment option (SUCRA 90.4%) followed by DB-MPFLR (SUCRA 84.6%). MR-plasty (SUCRA 30.3%) and MPR-plication(SUCRA 0.0%) ranked in the fourth and fifth positions behind the and VM-reefing(SUCRA 44.0%).

##### IKDC score

Five studies reported IKDC scores as one of the study outcomes. *I*^2^ = 24.4%, so we chosen the fixed effects model. Figure 2C show the MD and 95% CI of each surgery compared with DB-MPFLR. MR-plication (MD −18.01,95% CI, −20.05, −15.97), SB-MPFLR (MD −4.98, 95% CI, −6.34, −3.62) and VM-plasty (MD −8.71,95% CI, −12.82, −4.60) were inferior to DB-MPFLR. DB-MPFLR was at the top-ranking position (SUCRA 100.0%) shown in Table 3 followed by SB-MPFLR (SUCRA 65.8%). Two of the least effective treatments for IKDC Score were VM-plasty (SUCRA 34.2%) and MPR-plication (SUCRA 0.0%).

##### Redislocation

Nine studies reported redislocation as one of the study outcomes. *I*^2^ = 0.0%, so we chosen the fixed effects model. Figure 2D shows the OR and 95% CI of each surgery compared with DB-MPFLR. DB-MPFLR has no significant advantage over other surgical procedures in preventing redislocation, although it is the most likely intervention to be the best (SUCRA 67.8% shown in Table 3).

##### Recurrent instability

Eight studies reported recurrent instability as one of the study outcomes. *I*^2^ = 0.0%, so we chosen the fixed effects model. Figure 2E shows the OR and 95% CI of each surgery compared with DB-MPFLR. All the methods are not obviously inferior to DB-MPFLR. Even DB-MPFLR(SUCRA 70%) is less likely than VM-plasty(SUCRA 81.9%) to be the best intervention shown in shown in Table 3.

##### Subgroup

Since the clinical studies which reported Lyshlom and IKDC scores were all about recurrent patellar dislocation, we only conducted a subgroup analysis of Kujala score, redislocation, and recurrent instability. The results of the subgroup analysis were similar to those of the previous analysis. Detailed results are shown in Supplementary Appendix C and Table 3.

#### Publication bias

**Figure 3:**
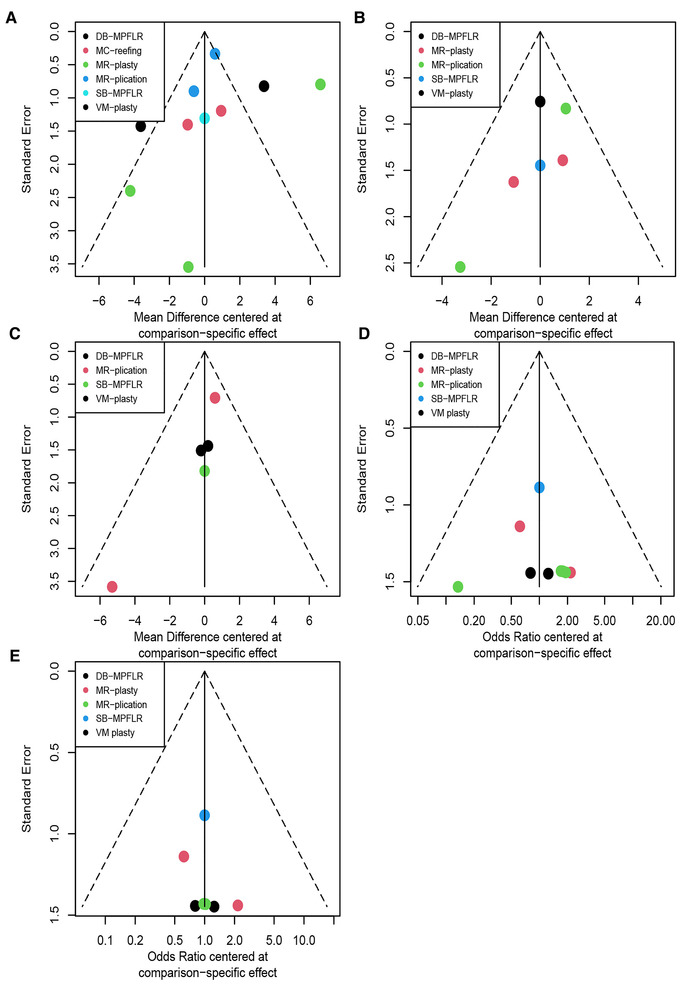
Adjusted funnel plot of different surgeries: (**A**) kujala score; (**B**) lysholm score; (**C**) IKDC score; (**D**) redislocation; (**E**) recurrent instability.

The comparison-adjusted funnel plots are displayed in Figures 3A–E. For the funnel plot of Kujala score, the outcomes showed obvious asymmetry, indicating a certain publication bias. The existence of points at the bottom of all funnel plots indicates that these outcomes all have small-study effects in the included studies.

#### Network plots

**Figure 4:**
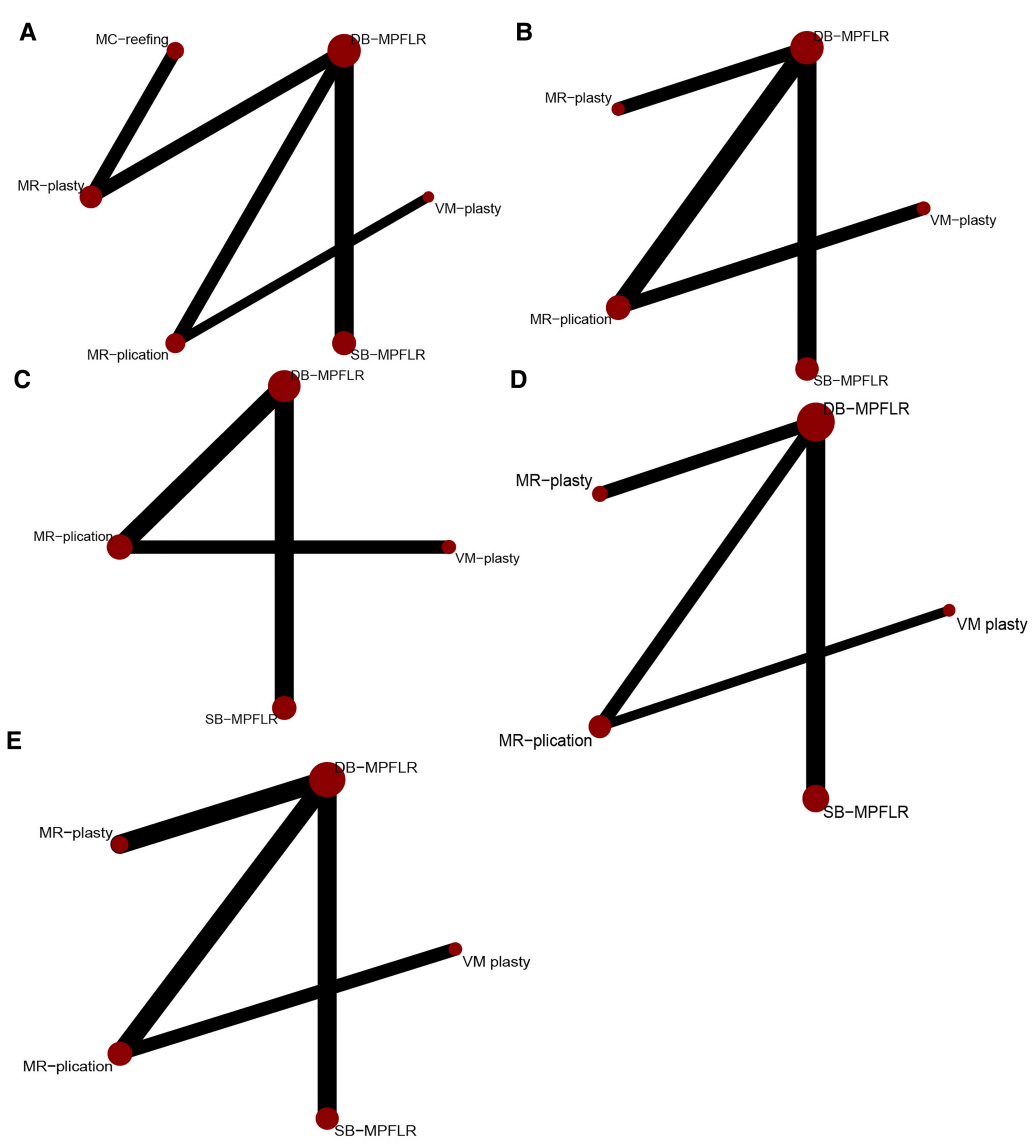
Network plot of treatment comparisons: (**A**) kujala score; (**B**) lysholm score; (**C**) IKDC score; (**D**) redislocation; (**E**) recurrent instability.

Trial network plots are shown in Figures 4A–E. The width of the line indicates the number of studies in which the two interventions are connected, and the size of the node indicates the number of patients receiving the intervention. Since there is no closed loop in each trial network plot, there is no inconsistency in NMA, and we only choose the consistency model.

#### CINeMA assessment

For the vast majority of interventions, the quality of evidence was “low” across the five outcome indicators. The comparisons of MC-reefing with DB-MPFLR and MR-plasty achieved very low

quality of evidence for the outcome of Kujala scores; the comparison of MR-plication with VM plasty achieved very low quality of evidence for the outcome of Recurrent instability (details were shown in Supplementary Appendix D).

#### Comparison of pair-wise meta-analysis and network meta-analysis

The comparison of pair-wise meta-analysis and network meta-analysis revealed that the results were generally consistent. This comparison between pair-wise meta-analysis and network meta-analysis confirmed the accuracy of the results.

## Discussion

The most important finding of this study was that DB-MPFLR is a fairly good method, which greatly improves function scores. In pairwise meta-analysis, DB-MPFLR shows great advantages in three outcomes. However, this conclusion requires careful consideration as it is highly heterogeneous, which may lead to the serious risk of bias. Also, we found the source of heterogeneity through meta-analysis: studies that compare DB-MPFLR and MR-plasty. For network meta-analysis, DB-MPFLR achieves the highest SURCA not only for patellar dislocation but also in recurrent patellar dislocation subgroup, except Lysholm score and recurrent instability. DB-MPFLR ranked second only to SB-MPFLR In Lysholm score and VM-plasty in recurrent instability. However, no significant difference was achieved in the prevention of dislocation and recurrent instability compared with other treatments.

A study has shown elevated TT-TG distance, trochlear dysplasia, patella alta and so that 92% of 175 patients had MPFL injury after a first time acute patellar dislocation (11). Injured sites include the femoral attachment and the patellar attachment (12). Moreover, a biomechanical study showed that MPFL provides approximately 60% of the inward binding resistance against lateral patella displacement (13). There is good reason to suspect that MPFLR, which can directly repair the medial patellar ligament, may yield higher functional outcomes. Based on this, there are many clinical studies of MPFLR for patellar dislocation to verify whether this is an appropriate method. In many studies, some have found that MPFLR is not superior to other soft tissue surgeries, but others have concluded that MPFLR does result in better functional scores than other surgeries (14–16). Therefore, our study collected high-quality RCTs and finally confirmed that MPFLR can obtain higher functional scores.

In adults, MPFLR has shown promising results, however, alternative MPFLR techniques are urgently needed for the treatment of recurrent patellar dislocation in children and adolescents with open growth plates. Several studies have developed a minimally invasive reconstruction of the MPFL through the insertion of the medial patellofemoral growth plate (17, 18).

For recurrent patellar dislocation, MPFL also achieved good results compared to other procedures in this study. However, other studies have found that risk factors for recurrent patellar dislocation include many bony structural abnormalities, such as elevated TT-TG distance, trochlear dysplasia, patella alta and so on (19). This means that simply repairing the inner soft tissue may not be enough. A clinical study reported that MPFLR combined with Tibial Tubercle Osteotomy obtained patellar kinematics and better functional scores compared with isolated MPFLR in the surgical treatment of recurrent patellar instability in patients with a TT-TG distance of 17–20 mm (20). Another study reported that the use of arthroscopic deepening tracheoplasty combined with MPFLR is a safe, reliable and reproducible surgical option, considering the stability of surgical results, knee function scores and patient satisfaction (21). However, a study showed that MPFLR combined with tuberosities transposition is not superior to isolated MPFLR on Kujala score and KOOS score (22). Moreover, A SurveyMonkey survey of 50 active surgeons in the International Patella Femur Study Group (IPSG) revealed inconsistent results on whether to perform bone surgeries for patients with recurrent patellar dislocation with bone abnormalities (23). So the jury is still out on whether bony surgery is needed.

The type of bundle reconstructed (SB or DB) is also a critical issue worth considering when surgeons conduct MPFLR on patients with patellar dislocation to restore normal patellar function. As previously mentioned, the MPFL is located in the second layer of soft tissue on the medial side of the knee joint and consists of two bundles, the inferior-straight bundle and the superior-oblique bundle respectively (4). A cadaver study reported that the attachment point of the patellofemoral ligament on the side of the patella is flexible and extends from the upper pole of the patella to the midpoint of the patella in a fan shape (24). As mentioned in the literature, the static constraint of medial soft tissue mainly depends on the inferior-straight bundle, while the dynamic constraint mainly depends on the superior-oblique bundle (4). SB-MPFLR which is reconstructed with only one bundle lost the normal patella-femoral ligament anatomy shape and thus lost binding to a larger area, while DB-MPFLR which is reconstructed with two bundles maximally mimicked the fan-shaped structure of the original patellofemoral ligament and thus gaining better constraint on the patella and being able to produce better clinical results. There have been many clinical trials of DB-MPFLR or SB-MPFLR, and the result is that DB-MPFLR is superior to SB-MPFLR on function scores or complications (25–27). This is exactly consistent with the results of our meta-analysis. A RCT of DB-MPFLR was conducted for graft morphology (28). One group was Y-shaped graft, and the other group was C-shaped graft (28). The result is that Y-graft technique was superior to C-graft technique in knee function scores for double bundle anatomic MPFL reconstruction, at a follow-up of at least 2 years (28). Although we have addressed many issues regarding MPFLR, including indications and bundle selection. But there are also many problems left for us, such as graft selection, fixation selection and so on. Therefore, more, larger scale and higher quality clinical studies are needed to find a better way to conduct DB-MPFLR.

Patella dislocation is not only a problem with the medial ligament, but also with the lateral ligament. Many studies have explored DBMPFLR in combination with other procedures. Chang Liu et al. compared the efficacy of different lateral ligament treatments combined with DB-MPFLR, and concluded that lateral retinaculum plasty would achieve better efficacy than lateral retinacular release (29).

The included clinical studies compared the outcomes of different types of surgeries for patellar dislocation (30). We also performed a subgroup analysis of recurrent patellar dislocation. There have been previous meta-analyses comparing MPFLR with other soft tissue surgeries, but either the quality of the included studies was low or there were few studies included. Of course, there are also studies comparing DB-MPFLR and SB-MPFLR, but they are only qualitative analyses. Therefore, this study conducted a meta-analysis of RCTs and provided high-quality evidence for the selection of the most effective methods of surgery.

There are some limitations. The validity of meta-analysis is closely related to the quality of the included studies and the number of studies between each direct comparison. In our study, the number of original studies between each comparison is small, and a comparison between MR-plication and VM-plasty contains only one original study. Many studies also had few outcome measures, with only the kujala score reported in all studies. Although RCTs are all included in our studies, the randomization methods and whether to allocate hidden are not described in some studies. And the level of evidence in this study is low or very low. In this study, we only discussed soft tissue surgery for patellar dislocation, while bone surgery was not included, which reduced the source of heterogeneity to a certain extent but also reduced the clinical applicability of this study.

## Conclusions

Our study demonstrates that MPFLR results in better functional scores than other soft tissue surgeries. Compared with SB-MPFLR, DB-MPFLR achieved higher scores in Kujala score and IKDC score, and lower scores only in Lysholm score.

## Data Availability

The raw data supporting the conclusions of this article will be made available by the authors, without undue reservation.
