# Supplementary material for: Surgical treatment of patellar dislocation: A network meta-analysis of randomized control trials and cohort studies
Source: Front Surg. 2023 Mar 30;10:1003796. doi: 10.3389/fsurg.2023.1003796 (PMC10097987; doi:10.3389/fsurg.2023.1003796)
Supplement: Supplementary file 1 [file Datasheet1.docx]

Appendix

Appendix A. Search strategy.（p2-3）

Appendix B. Risk of bias assessment.（p4-5）

Appendix C. Sub-group analysis.（p6）

Appendix D. Confidence in network meta analysis (CINeMA)（p7-8）

Appendix E. PRISMA 2020 checklist.（p 9-12）

**Appendix A.** Search strategy.

**Electronic Search Strategy in Pubmed**

| 1."patellar dislocation"[MeSH Terms] |
| --- |
| 2."patella"[MeSH Terms] AND "joint dislocations"[MeSH Terms] |
| 3.("dislocat*"[Title/Abstract] OR "sublux*"[Title/Abstract] OR "instability"[Title/Abstract]) AND "patell*"[Title/Abstract] |
| 4.1 OR 2 OR 3 |
| 5.("medial patellofemoral ligament"[Title/Abstract] OR "MPFL"[Title/Abstract]) AND ("reconstruction"[Title/Abstract] OR "reconstructions"[Title/Abstract]) |
| 6.("single"[Title/Abstract] OR "SB"[Title/Abstract]) AND ("double"[Title/Abstract] OR "DB"[Title/Abstract]) |
| 7.5 AND 6 |
| 8."plication"[Title/Abstract] OR "plasty"[Title/Abstract] OR "reefing"[Title/Abstract] |
| 9.5 AND 8 |
| 10.("plasty"[Title/Abstract] OR "reefing"[Title/Abstract]) AND "plication"[Title/Abstract] |
| 11."plasty"[Title/Abstract] AND "reefing"[Title/Abstract] |
| 12.7 OR 9 OR 10 OR 11 |
| 13.4 AND 12 |

**Electronic Search Strategy in Web of Science**

| 1 | ((((TS=(patell*)) OR TS=(dislocat*)) OR TS=(sublux*)) OR TS=(instability)) AND TS=(patell*) |
| --- | --- |
| 2 | (TS=(medial patellofemoral ligament)) OR TS=(MPFL) |
| 3 | (TS=(reconstruction)) OR TS=(reconstructions) |
| 4 | #3 AND #2 |
| 5 | (TS=(single)) OR TS=(SB) |
| 6 | (TS=(double)) OR TS=(DB) |
| 7 | #6 AND #5 |
| 8 | #7 AND #4 |
| 9 | ((TS=(plication)) OR TS=(plasty)) OR TS=(reefing) |
| 10 | #9 AND #4 |
| 11 | (TS=(plasty)) OR TS=(reefing) |
| 12 | TS=(plication) |
| 13 | #12 AND #11 |
| 14 | (TS=(plasty)) AND TS=(reefing) |
| 15 | #14 OR #13 OR #10 OR #8 |
| 16 | #15 AND #1 |

**Electronic Search Strategy in Cochrane Central Register of Controlled Trials**

| #1 | MeSH descriptor: [Patellar Dislocation] explode all trees |
| --- | --- |
| #2 | MeSH descriptor: [Patella] explode all trees |
| #3 | MeSH descriptor: [Joint Dislocations] explode all trees |
| #4 | #2 AND #3 |
| #5 | (dislocat*):ti,ab,kw OR (sublux*):ti,ab,kw OR (instability):ti,ab,kw |
| #6 | (patell*):ti,ab,kw |
| #7 | #5 AND #6 |
| #8 | #1 OR #4 OR #7 |
| #9 | (medial patellofemoral ligament):ti,ab,kw OR (MPFL):ti,ab,kw |
| #10 | (reconstruction):ti,ab,kw OR (reconstructions):ti,ab,kw |
| #11 | #9 AND #10 |
| #12 | (single):ti,ab,kw OR (SB):ti,ab,kw |
| #13 | (double):ti,ab,kw OR (DB):ti,ab,kw |
| #14 | #12 AND #13 |
| #15 | #11 AND #14 |
| #16 | (plication):ti,ab,kw OR (plasty):ti,ab,kw OR (reefing):ti,ab,kw |
| #17 | #11 AND #16 |
| #18 | (plasty):ti,ab,kw OR (reefing):ti,ab,kw |
| #19 | (plication):ti,ab,kw |
| #20 | #18 AND #19 |
| #21 | (plasty):ti,ab,kw AND (reefing):ti,ab,kw |
| #22 | #15 OR #17 OR #20 OR #21 |
| #23 | #22 AND #8 |

**Electronic Search Strategy in Embase**

| #21 | #6 AND #20 |
| --- | --- |
| #20 | #13 OR #15 OR #18 OR #19 |
| #19 | plasty:ab,ti AND reefing:ab,ti |
| #18 | #16 AND #17 |
| #17 | plication:ab,ti |
| #16 | plasty:ab,ti OR reefing:ab,ti |
| #15 | #9 AND #14 |
| #14 | plication:ab,ti OR plasty:ab,ti OR reefing:ab,ti |
| #13 | #9 AND #12 |
| #12 | #10 AND #11 |
| #11 | double:ab,ti OR db:ab,ti |
| #10 | single:ab,ti OR sb:ab,ti |
| #9 | #7 AND #8 |
| #8 | reconstruction:ab,ti OR reconstructions:ab,ti |
| #7 | 'medial patellofemoral ligament':ab,ti OR mpfl:ab,ti |
| #6 | #1 OR #2 OR #5 |
| #5 | #3 AND #4 |
| #4 | patell*:ab,ti |
| #3 | dislocat*:ab,ti OR sublux:ab,ti OR instability:ab,ti |
| #2 | 'patella'/exp AND 'joint dislocation'/exp |
| #1 | 'patellar dislocation'/exp |

**Appendix B. Risk of bias assessment.**


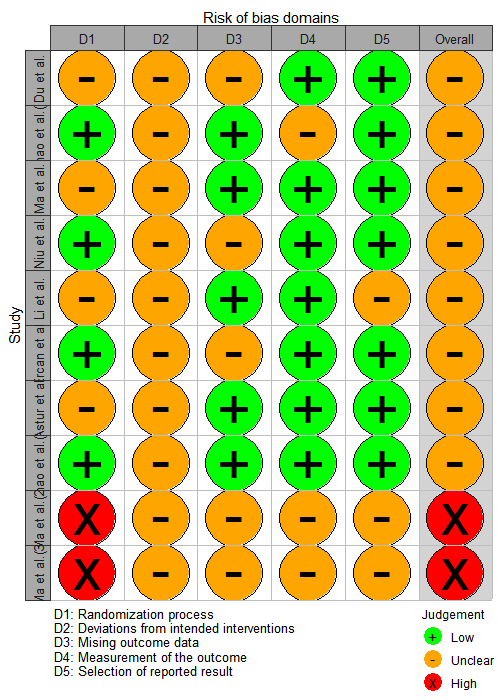


(a)The data on the risk of bias assessment for each study.


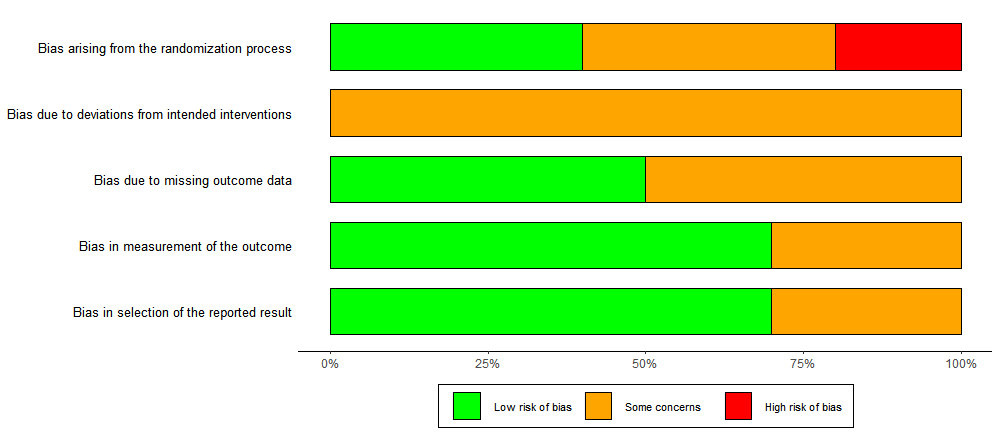


(b)Summary of bias risk assessment.

| (c) The Newcastle-Ottawa Quality Assessment Scale for Included Cohort Studies | | | | |
| --- | --- | --- | --- | --- |
| Lead Author (Year) | Selection of the Study Groups | Comparability of the Groups | Outcome | Total Scores |
| Feng (2020) | 3 | 2 | 2 | 7 |
| Wang (2013) | 3 | 2 | 3 | 8 |

**Appendix C. Sub-group analysis.**


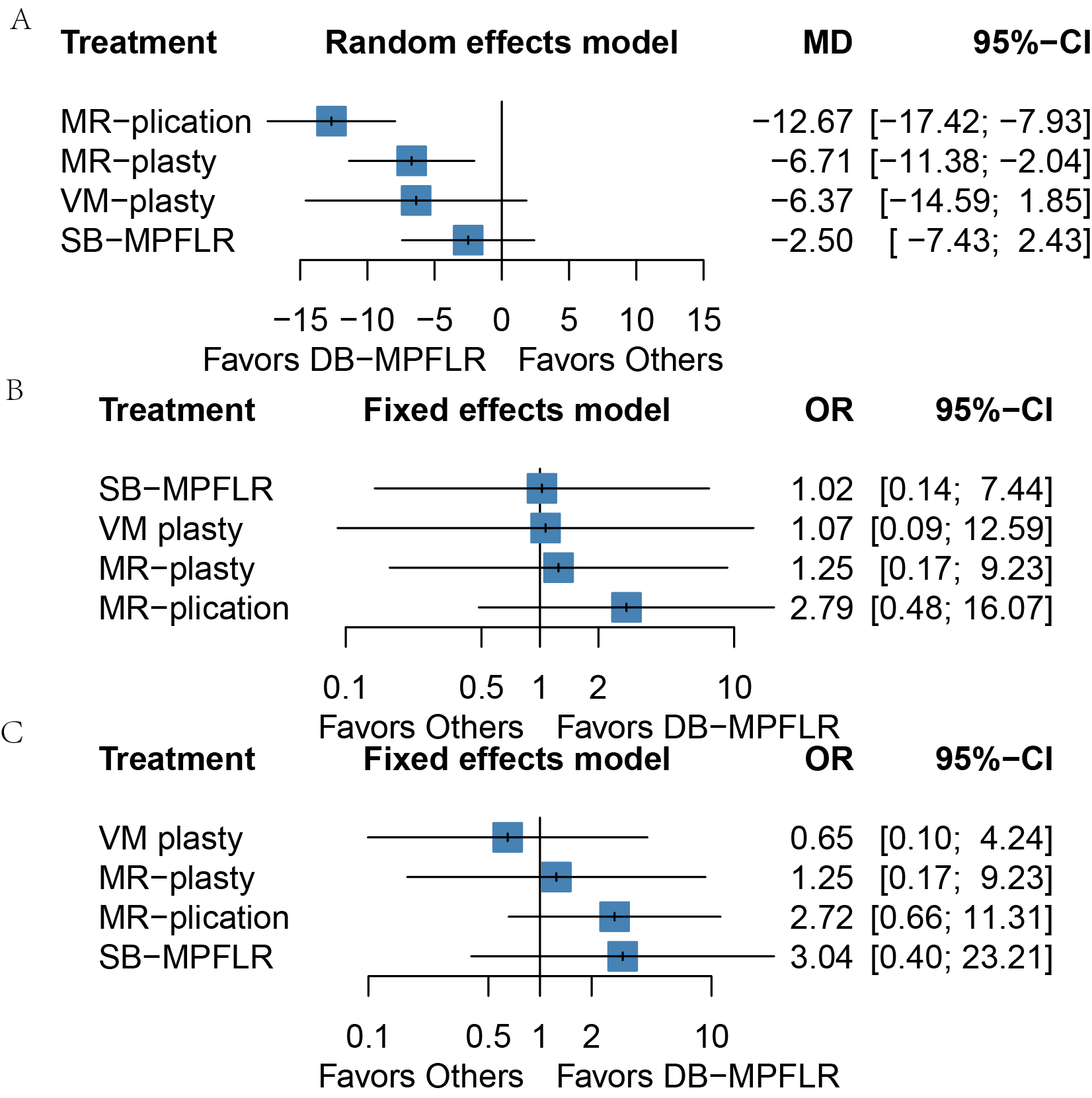


Forest plot of Sub-group analysis: A: Kujala score; B: Redislocation; C: Recurrent instability

**Appendix D.** Confidence in network meta analysis (CINeMA)

CINeMA tool for the results of the effect of different surgeries on Kujala scores

| Comparison | Number of studies | Within-study bias | Reporting bias | Indirectness | Imprecision | Heterogeneity | Incoherence | Confidence rating | Reason(s) for downgrading |
| --- | --- | --- | --- | --- | --- | --- | --- | --- | --- |
| DB-MPFLR:MR-plasty | 2 | Some concerns | Low risk | No concerns | No concerns | Major concerns | Major concerns | Low | ["Within-study bias","Heterogeneity","Incoherence"] |
| DB-MPFLR:MR-plication | 2 | Some concerns | Low risk | No concerns | No concerns | Major concerns | Major concerns | Low | ["Within-study bias","Heterogeneity","Incoherence"] |
| DB-MPFLR:SB-MPFLR | 4 | Some concerns | Low risk | No concerns | No concerns | Major concerns | Major concerns | Low | ["Within-study bias","Heterogeneity","Incoherence"] |
| MC-reefing:MR-plasty | 2 | Major concerns | Low risk | No concerns | Major concerns | No concerns | Major concerns | Very low | ["Within-study bias","Imprecision","Incoherence"] |
| MR-plication:VM-plasty | 1 | Some concerns | Low risk | No concerns | Major concerns | No concerns | Major concerns | Low | ["Within-study bias","Imprecision","Incoherence"] |
| DB-MPFLR:MC-reefing | 0 | Major concerns | Low risk | No concerns | No concerns | Major concerns | Major concerns | Very low | ["Within-study bias","Heterogeneity","Incoherence"] |
| DB-MPFLR:VM-plasty | 0 | Some concerns | Low risk | No concerns | Major concerns | No concerns | Major concerns | Low | ["Within-study bias","Imprecision","Incoherence"] |
| MC-reefing:MR-plication | 0 | Some concerns | Low risk | No concerns | Major concerns | No concerns | Major concerns | Low | ["Within-study bias","Imprecision","Incoherence"] |
| MC-reefing:SB-MPFLR | 0 | Some concerns | Low risk | No concerns | Major concerns | No concerns | Major concerns | Low | ["Within-study bias","Imprecision","Incoherence"] |
| MC-reefing:VM-plasty | 0 | Some concerns | Low risk | No concerns | Major concerns | No concerns | Major concerns | Low | ["Within-study bias","Imprecision","Incoherence"] |
| MR-plasty:MR-plication | 0 | Some concerns | Low risk | No concerns | Major concerns | No concerns | Major concerns | Low | ["Within-study bias","Imprecision","Incoherence"] |
| MR-plasty:SB-MPFLR | 0 | Some concerns | Low risk | No concerns | Major concerns | No concerns | Major concerns | Low | ["Within-study bias","Imprecision","Incoherence"] |
| MR-plasty:VM-plasty | 0 | Some concerns | Low risk | No concerns | Major concerns | No concerns | Major concerns | Low | ["Within-study bias","Imprecision","Incoherence"] |
| MR-plication:SB-MPFLR | 0 | Some concerns | Low risk | No concerns | Major concerns | No concerns | Major concerns | Low | ["Within-study bias","Imprecision","Incoherence"] |
| SB-MPFLR:VM-plasty | 0 | Some concerns | Low risk | No concerns | Major concerns | No concerns | Major concerns | Low | ["Within-study bias","Imprecision","Incoherence"] |

CINeMA tool for the results of the effect of different surgeries on Lysholm scores

| Comparison | Number of studies | Within-study bias | Reporting bias | Indirectness | Imprecision | Heterogeneity | Incoherence | Confidence rating | Reason(s) for downgrading |
| --- | --- | --- | --- | --- | --- | --- | --- | --- | --- |
| DB-MPFLR:MR-plasty | 1 | Some concerns | Low risk | No concerns | No concerns | Major concerns | Major concerns | Low | ["Within-study bias","Heterogeneity","Incoherence"] |
| DB-MPFLR:MR-plication | 2 | Some concerns | Low risk | No concerns | No concerns | Major concerns | Major concerns | Low | ["Within-study bias","Heterogeneity","Incoherence"] |
| DB-MPFLR:SB-MPFLR | 2 | Some concerns | Low risk | No concerns | Major concerns | No concerns | Major concerns | Low | ["Within-study bias","Imprecision","Incoherence"] |
| MR-plication:VM-plasty | 1 | Some concerns | Low risk | No concerns | No concerns | Major concerns | Major concerns | Low | ["Within-study bias","Heterogeneity","Incoherence"] |
| DB-MPFLR:VM-plasty | 0 | Some concerns | Low risk | No concerns | No concerns | Major concerns | Major concerns | Low | ["Within-study bias","Heterogeneity","Incoherence"] |
| MR-plasty:MR-plication | 0 | Some concerns | Low risk | No concerns | No concerns | Major concerns | Major concerns | Low | ["Within-study bias","Heterogeneity","Incoherence"] |
| MR-plasty:SB-MPFLR | 0 | Some concerns | Low risk | No concerns | No concerns | Major concerns | Major concerns | Low | ["Within-study bias","Heterogeneity","Incoherence"] |
| MR-plasty:VM-plasty | 0 | Some concerns | Low risk | No concerns | Major concerns | No concerns | Major concerns | Low | ["Within-study bias","Imprecision","Incoherence"] |
| MR-plication:SB-MPFLR | 0 | Some concerns | Low risk | No concerns | No concerns | Major concerns | Major concerns | Low | ["Within-study bias","Heterogeneity","Incoherence"] |
| SB-MPFLR:VM-plasty | 0 | Some concerns | Low risk | No concerns | No concerns | Major concerns | Major concerns | Low | ["Within-study bias","Heterogeneity","Incoherence"] |

CINeMA tool for the results of the effect of different surgeries on IKDC scores

| Comparison | Number of studies | Within-study bias | Reporting bias | Indirectness | Imprecision | Heterogeneity | Incoherence | Confidence rating | Reason(s) for downgrading |
| --- | --- | --- | --- | --- | --- | --- | --- | --- | --- |
| DB-MPFLR:MR-plication | 2 | Some concerns | Low risk | No concerns | No concerns | Major concerns | Major concerns | Low | ["Within-study bias","Heterogeneity","Incoherence"] |
| DB-MPFLR:SB-MPFLR | 2 | Some concerns | Low risk | No concerns | No concerns | Major concerns | Major concerns | Low | ["Within-study bias","Heterogeneity","Incoherence"] |
| MR-plication:VM-plasty | 1 | Some concerns | Low risk | No concerns | No concerns | Major concerns | Major concerns | Low | ["Within-study bias","Incoherence"] |
| DB-MPFLR:VM-plasty | 0 | Some concerns | Low risk | No concerns | No concerns | Major concerns | Major concerns | Low | ["Within-study bias","Heterogeneity","Incoherence"] |
| MR-plication:SB-MPFLR | 0 | Some concerns | Low risk | No concerns | No concerns | Major concerns | Major concerns | Low | ["Within-study bias","Heterogeneity","Incoherence"] |
| SB-MPFLR:VM-plasty | 0 | Some concerns | Low risk | No concerns | Major concerns | No concerns | Major concerns | Low | ["Within-study bias","Imprecision","Incoherence"] |

CINeMA tool for the results of the effect of different surgeries on Redislocation

| Comparison | Number of studies | Within-study bias | Reporting bias | Indirectness | Imprecision | Heterogeneity | Incoherence | Confidence rating | Reason(s) for downgrading |
| --- | --- | --- | --- | --- | --- | --- | --- | --- | --- |
| DB-MPFLR:MR-plasty | 2 | Some concerns | Low risk | No concerns | Major concerns | No concerns | Major concerns | Low | ["Within-study bias","Imprecision","Incoherence"] |
| DB-MPFLR:MR-plication | 2 | Some concerns | Low risk | No concerns | Major concerns | No concerns | Major concerns | Low | ["Within-study bias","Imprecision","Incoherence"] |
| DB-MPFLR:SB-MPFLR | 4 | Some concerns | Low risk | No concerns | Major concerns | No concerns | Major concerns | Low | ["Within-study bias","Imprecision","Incoherence"] |
| MR-plication:VM plasty | 1 | Some concerns | Low risk | No concerns | Major concerns | No concerns | Major concerns | Low | ["Within-study bias","Imprecision","Incoherence"] |
| DB-MPFLR:VM plasty | 0 | Some concerns | Low risk | No concerns | Major concerns | No concerns | Major concerns | Low | ["Within-study bias","Imprecision","Incoherence"] |
| MR-plasty:MR-plication | 0 | Some concerns | Low risk | No concerns | Major concerns | No concerns | Major concerns | Low | ["Within-study bias","Imprecision","Incoherence"] |
| MR-plasty:SB-MPFLR | 0 | Some concerns | Low risk | No concerns | Major concerns | No concerns | Major concerns | Low | ["Within-study bias","Imprecision","Incoherence"] |
| MR-plasty:VM plasty | 0 | Some concerns | Low risk | No concerns | Major concerns | No concerns | Major concerns | Low | ["Within-study bias","Imprecision","Incoherence"] |
| MR-plication:SB-MPFLR | 0 | Some concerns | Low risk | No concerns | Major concerns | No concerns | Major concerns | Low | ["Within-study bias","Imprecision","Incoherence"] |
| SB-MPFLR:VM plasty | 0 | Some concerns | Low risk | No concerns | Major concerns | No concerns | Major concerns | Low | ["Within-study bias","Imprecision","Incoherence"] |

CINeMA tool for the results of the effect of different surgeries on Recurrent instability

| Comparison | Number of studies | Within-study bias | Reporting bias | Indirectness | Imprecision | Heterogeneity | Incoherence | Confidence rating | Reason(s) for downgrading |
| --- | --- | --- | --- | --- | --- | --- | --- | --- | --- |
| DB-MPFLR:MR-plasty | 2 | Some concerns | Low risk | No concerns | Major concerns | No concerns | Major concerns | Low | ["Within-study bias","Imprecision","Incoherence"] |
| DB-MPFLR:MR-plication | 1 | Some concerns | Low risk | No concerns | Major concerns | No concerns | Major concerns | Low | ["Within-study bias","Imprecision","Incoherence"] |
| DB-MPFLR:SB-MPFLR | 4 | Some concerns | Low risk | No concerns | No concerns | Major concerns | Major concerns | low | ["Within-study bias","Heterogeneity","Incoherence"] |
| MR-plication:VM plasty | 1 | Some concerns | Low risk | No concerns | No concerns | Major concerns | Major concerns | Very low | ["Within-study bias","Heterogeneity","Incoherence"] |
| DB-MPFLR:VM plasty | 0 | Some concerns | Low risk | No concerns | Major concerns | No concerns | Major concerns | Low | ["Within-study bias","Imprecision","Incoherence"] |
| MR-plasty:MR-plication | 0 | Some concerns | Low risk | No concerns | Major concerns | No concerns | Major concerns | Low | ["Within-study bias","Imprecision","Incoherence"] |
| MR-plasty:SB-MPFLR | 0 | Some concerns | Low risk | No concerns | Major concerns | No concerns | Major concerns | Low | ["Within-study bias","Imprecision","Incoherence"] |
| MR-plasty:VM plasty | 0 | Some concerns | Low risk | No concerns | Major concerns | No concerns | Major concerns | Low | ["Within-study bias","Imprecision","Incoherence"] |
| MR-plication:SB-MPFLR | 0 | Some concerns | Low risk | No concerns | Major concerns | No concerns | Major concerns | Low | ["Within-study bias","Imprecision","Incoherence"] |
| SB-MPFLR:VM plasty | 0 | Some concerns | Low risk | No concerns | Major concerns | No concerns | Major concerns | Low | ["Within-study bias","Imprecision","Incoherence"] |

**Appendix E. PRISMA 2020 checklist.**

| **Section and Topic** | **Item #** | **Checklist item** | **Location where item is reported** |
| --- | --- | --- | --- |
| **TITLE** | | |  |
| Title | 1 | Identify the report as a systematic review. | Page 1 |
| **ABSTRACT** | | |  |
| Abstract | 2 | See the PRISMA 2020 for Abstracts checklist. | Page 2 |
| **INTRODUCTION** | | |  |
| Rationale | 3 | Describe the rationale for the review in the context of existing knowledge. | Page 3 |
| Objectives | 4 | Provide an explicit statement of the objective(s) or question(s) the review addresses. | Page 3 |
| **METHODS** | | |  |
| Eligibility criteria | 5 | Specify the inclusion and exclusion criteria for the review and how studies were grouped for the syntheses. | Page 4 |
| Information sources | 6 | Specify all databases, registers, websites, organisations, reference lists and other sources searched or consulted to identify studies. Specify the date when each source was last searched or consulted. | Page 4 |
| Search strategy | 7 | Present the full search strategies for all databases, registers and websites, including any filters and limits used. | Appendix A |
| Selection process | 8 | Specify the methods used to decide whether a study met the inclusion criteria of the review, including how many reviewers screened each record and each report retrieved, whether they worked independently, and if applicable, details of automation tools used in the process. | Page 4 |
| Data collection process | 9 | Specify the methods used to collect data from reports, including how many reviewers collected data from each report, whether they worked independently, any processes for obtaining or confirming data from study investigators, and if applicable, details of automation tools used in the process. | Page 5 |
| Data items | 10a | List and define all outcomes for which data were sought. Specify whether all results that were compatible with each outcome domain in each study were sought (e.g. for all measures, time points, analyses), and if not, the methods used to decide which results to collect. | Page 5 |
|  | 10b | List and define all other variables for which data were sought (e.g. participant and intervention characteristics, funding sources). Describe any assumptions made about any missing or unclear information. | Page 5 |
| Study risk of bias assessment | 11 | Specify the methods used to assess risk of bias in the included studies, including details of the tool(s) used, how many reviewers assessed each study and whether they worked independently, and if applicable, details of automation tools used in the process. | Page 5 |
| Effect measures | 12 | Specify for each outcome the effect measure(s) (e.g. risk ratio, mean difference) used in the synthesis or presentation of results. | Page 5,6 |
| Synthesis methods | 13a | Describe the processes used to decide which studies were eligible for each synthesis (e.g. tabulating the study intervention characteristics and comparing against the planned groups for each synthesis (item #5)). | Page 5,6 |
|  | 13b | Describe any methods required to prepare the data for presentation or synthesis, such as handling of missing summary statistics, or data conversions. | Page 5,6 |
|  | 13c | Describe any methods used to tabulate or visually display results of individual studies and syntheses. | Page 5,6 |
|  | 13d | Describe any methods used to synthesize results and provide a rationale for the choice(s). If meta-analysis was performed, describe the model(s), method(s) to identify the presence and extent of statistical heterogeneity, and software package(s) used. | Page 5,6 |
|  | 13e | Describe any methods used to explore possible causes of heterogeneity among study results (e.g. subgroup analysis, meta-regression). | Page 5,6 |
|  | 13f | Describe any sensitivity analyses conducted to assess robustness of the synthesized results. | Page 5,6 |
| Reporting bias assessment | 14 | Describe any methods used to assess risk of bias due to missing results in a synthesis (arising from reporting biases). | Page 6 |
| Certainty assessment | 15 | Describe any methods used to assess certainty (or confidence) in the body of evidence for an outcome. | Page 6 |
| **RESULTS** | | |  |
| Study selection | 16a | Describe the results of the search and selection process, from the number of records identified in the search to the number of studies included in the review, ideally using a flow diagram. | Page 7 |
|  | 16b | Cite studies that might appear to meet the inclusion criteria, but which were excluded, and explain why they were excluded. | Figure 1 |
| Study characteristics | 17 | Cite each included study and present its characteristics. | Table 1 and Page 7 |
| Risk of bias in studies | 18 | Present assessments of risk of bias for each included study. | Appendix B and Page 8 |
| Results of individual studies | 19 | For all outcomes, present, for each study: (a) summary statistics for each group (where appropriate) and (b) an effect estimate and its precision (e.g. confidence/credible interval), ideally using structured tables or plots. | Page 8,9 and Table 2 |
| Results of syntheses | 20a | For each synthesis, briefly summarise the characteristics and risk of bias among contributing studies. | Page 8,9 ,10 |
|  | 20b | Present results of all statistical syntheses conducted. If meta-analysis was done, present for each the summary estimate and its precision (e.g. confidence/credible interval) and measures of statistical heterogeneity. If comparing groups, describe the direction of the effect. | Page 8,9 ,10 and  Appendix C |
|  | 20c | Present results of all investigations of possible causes of heterogeneity among study results. | Page 9 ,10 and  Appendix C |
|  | 20d | Present results of all sensitivity analyses conducted to assess the robustness of the synthesized results. | Page 8,9 ,10 and  Appendix C |
| Reporting biases | 21 | Present assessments of risk of bias due to missing results (arising from reporting biases) for each synthesis assessed. | Appendix B |
| Certainty of evidence | 22 | Present assessments of certainty (or confidence) in the body of evidence for each outcome assessed. | Page 11 AND Appendix D |
| **DISCUSSION** | | |  |
| Discussion | 23a | Provide a general interpretation of the results in the context of other evidence. | Page 12 |
|  | 23b | Discuss any limitations of the evidence included in the review. | Page 14,15 |
|  | 23c | Discuss any limitations of the review processes used. | Page 15 |
|  | 23d | Discuss implications of the results for practice, policy, and future research. | Page 14,15 |
| **OTHER INFORMATION** | | |  |
| Registration and protocol | 24a | Provide registration information for the review, including register name and registration number, or state that the review was not registered. | not registered |
|  | 24b | Indicate where the review protocol can be accessed, or state that a protocol was not prepared. | protocol was not prepared |
|  | 24c | Describe and explain any amendments to information provided at registration or in the protocol. | NO |
| Support | 25 | Describe sources of financial or non-financial support for the review, and the role of the funders or sponsors in the review. | Page 20 |
| Competing interests | 26 | Declare any competing interests of review authors. | Page 20 |
| Availability of data, code and other materials | 27 | Report which of the following are publicly available and where they can be found: template data collection forms; data extracted from included studies; data used for all analyses; analytic code; any other materials used in the review. | Page 20 |
